# Astrocyte Elevated Gene-1/Metadherin (AEG-1/MTDH): A Promising Molecular Marker and Therapeutic Target for Hepatocellular Carcinoma

**DOI:** 10.3390/cancers17081375

**Published:** 2025-04-21

**Authors:** Eva Davis, Ali Gawi Ermi, Devanand Sarkar

**Affiliations:** 1 Department of Microbiology and Immunology, Virginia Commonwealth University, Richmond, VA 23298, USA; davisep@vcu.edu; 2 Department of Cellular, Molecular and Genetic Medicine, Virginia Commonwealth University, Richmond, VA 23298, USA; ali.gawiermi@vcuhealth.org; 3 Department of Cellular, Molecular and Genetic Medicine, Massey Comprehensive Cancer Center, Virginia Commonwealth University, Richmond, VA 23298, USA

**Keywords:** astrocyte elevated gene-1 (AEG-1), metadherin (MTDH), hepatocellular carcinoma (HCC)

## Abstract

Hepatocellular carcinoma (HCC) is one of the leading causes of cancer-related deaths. The 5-year survival rate has been estimated to be less than 20% while its incidence rates have more than tripled since the 1980s. Astrocyte elevated gene-1/Metadherin (AEG-1/MTDH) has been demonstrated to have an influential role in HCC progression and the development of an aggressive phenotype. AEG-1 has been shown to be upregulated in many cancers, including HCC. Studies have shown that it plays a crucial role in the proliferation, invasion and metastasis, and evasion of apoptosis in HCC. Its relationship with proteins and pathways, such as MYC, SND1, PI3K/AKT, and other signaling pathways demonstrates its pertinent role in oncogenic development and relevance as a biomarker and therapeutic target. Recent studies have shown that AEG-1 is present in tumor tissues, and the anti-AEG-1 antibody is detected in the blood of cancer patients, demonstrating its viability as a diagnostic/prognostic marker. This review paper shines light on recent findings regarding the molecular implications of AEG-1, with emphasis on its role of regulating metabolic dysfunction-associated steatohepatitis (MASH), a key predisposing factor for HCC, new treatment strategies targeting AEG-1, and challenges associated with analyzing this intriguing molecule.

## Simple Summary

Astrocyte elevated gene-1/Metadherin (*AEG-1/MTDH*) is a gene known for its overexpression in various cancers, including hepatocellular carcinoma (HCC). It plays a key role in aggressive tumorigenesis through an array of processes and mechanisms, and is associated with chemoresistance and immune suppressive oncogenic pathways. AEG-1 shows clear promise as a prognostic marker and therapeutic target. In this review, we provide a clear and concise description of AEG-1′s background, its implications in HCC development, and the importance of AEG-1-targeted therapeutics.

## 1. Introduction

### 1.1. Hepatocellular Carcinoma: Epidemiology and Risk Factors

Cancer accounts for 16.8% of all deaths and 22.8% of deaths from noncommunicable diseases, making it the second leading cause of mortality globally [1]. As the saying goes, cancer is as old as the human race, and a description of breast cancer has been found on Egyptian papyrus from as early as 3000 BC [2]. The first historical description of liver cancer was found in the book *De Medicina* by the Greek physician Aulus Celsus (25 BC–50 AD) [3]. The main cell types in the liver are hepatocytes and cholangiocytes, from which primary liver cancer originates. Hepatocytes give rise to hepatocellular carcinoma (HCC), while intrahepatic cholangiocarcinoma (ICC) develops from cholangiocytes. However, some HCCs and ICCs may arise from cancer stem cells or hepatic progenitor cells [4]. HCC accounts for 75–85% of primary liver cancer cases and ranks as the third leading cause of cancer-related deaths and the sixth most predominant cancer globally [1]. HCC tends to be an aggressive and lethal cancer, with a five-year survival rate of less than 20% and a recurrence rate of up to 88% [1]. According to Global Cancer Statistics, in 2022, 865,269 new cases of primary liver cancer were diagnosed with 757,948 deaths occurring [1]. In the US, for 2024, it was estimated that 41,630 new HCC cases occurred, of which 29,840 were expected to be terminal [5]. HCC is now becoming one of the fastest rising causes of death in the United States, drastically increasing the need to identify potential therapeutic targets [5]. In most cases, there is an underlying cause that induces chronic hepatitis which culminates in HCC. The most common cause of HCC is viral hepatitis arising from hepatitis B virus (HBV) or hepatitis C virus (HCV) infection [6]. An increasingly important determinant of HCC now is metabolic dysfunction-associated steatotic liver disease (MASLD), a consequence of obesity, the incidence of which is increasing alarmingly all over the world [7,8]. Additional causes include chronic alcoholism, aflatoxins, α1-antitrypsin deficiency, autoimmune hepatitis, porphyrias, and Wilson disease [9,10]. In all of these cases, there is an injury to the hepatocytes resulting in simultaneous death and compensatory proliferation, and the release of pro-inflammatory cytokines from the damaged hepatocytes which stimulate inflammation, as well as fibrosis, by activating the hepatic stellate cells. When the process continues for decades, there is cirrhosis as well as DNA damage causing mutation, which creates a favorable milieu for HCC development [9].

### 1.2. HCC: Treatment Strategy

The treatment strategies for HCC depend on the stage of the disease. The early and intermediate stages of HCC, characterized by single or multinodular tumors with preserved liver function, can be managed by medical interventions such as liver transplantation, curative resection, transarterial chemoembolization (TACE), radiofrequency ablation (RFA), and radioembolization, which can provide an expected survival of >5 years for early-stage HCC and >2.5 years for intermediate-stage disease [11,12]. However, HCC at early and intermediate stages presents with vague symptoms, so most HCC cases are diagnosed at advanced stages, characterized by portal vein invasion and/or extrahepatic metastasis with the preservation of liver function [12]. Due to the prevalence of pre-existing liver damage or cirrhosis, accompanied by the intrinsic resistance of HCC cells toward conventional treatments, such as chemo- and/or radiotherapy, advanced stage HCC is often coupled with poor prognosis. [11,13]. A combination of two antibodies, the anti-programmed cell death ligand 1 (anti-PD-L1; atezolizumab) and anti-vascular endothelial growth factor (anti-VEGF; bevacizumab) antibodies, has proven to be the most effective FDA-approved first-line treatment for advanced non-resectable HCC with an overall response rate of 27% [12,14,15]. A combination of anti-PD-L1 (durvalumab) and anti-Cytotoxic T-Lymphocyte-Associated Protein 4 (anti-CTLA-4; tremelimumab) antibodies is additional first-line treatment for advanced HCC [12,16]. In case immunotherapy is not feasible, systemic therapy with tyrosine kinase inhibitors (TKIs, such as sorafenib or lenvatinib) is used for advanced HCC as a first-line treatment [12]. TKIs, such as regorafenib and cabozantinib, and the anti-VEGF receptor 2 (anti-VEGFR2) antibody (ramucirumab) are used as second- or third-line treatments for advanced HCC [12]. All these approaches provide an expected survival of ~2 years and TKIs provide a median overall survival expectancy of only 10.4 months with the development of drug resistance in 100% of cases [12]. Terminal HCC with end-stage liver function is managed only by palliative treatment with an expected survival of only 3 months [12]. For this reason, it is vital to determine therapeutic targets that have the potential to significantly impact the survival rates of HCC patients over and beyond what is achievable with current treatment strategies.

### 1.3. HCC: Diagnostic and Prognostic Markers

De-differentiation is a hallmark of cancer characterized by the re-expression of onco-fetal proteins. Alpha-fetoprotein (AFP) is a glycoprotein produced by the yolk sac and the liver during fetal life and it functions in immune modulation [17]. Among the most clinically used diagnostic markers for HCC, AFP stands as the most commonly used serum biomarker. Despite its moderate sensitivity being ~40–60% and its high specificity of ~80–90%, AFP levels above 200 ng/mL are a strong sign of HCC [18]. However, high or above-normal levels can also occur in chronic liver diseases like cirrhosis and hepatitis. Lens culinaris agglutinin reactive AFP (AFP-L3), a glycoform of AFP, provides additional specificity for HCC and is particularly useful in distinguishing aggressive tumors [19]. Another serum biomarker is des-γ-carboxy prothrombin (DCP), also known as protein induced by vitamin K absence-II (PIVKA-II), an abnormal form of prothrombin produced by malignant hepatocytes. DCP has been demonstrated to have a better specificity for HCC than AFP, particularly in distinguishing tumors with vascular invasion [20].

Tissue-based biomarkers, such as glypican-3 (GPC3), a cell surface glycoprotein, show high specificity for HCC and are frequently used in immunohistochemistry (IHC) for histopathological diagnosis [21]. Despite their lack of establishment within clinical practice, emerging biomarkers, such as circulating microRNAs (miR-21, miR-122, miR-221, miR-224, miR-29a) and circulating tumor DNA (ctDNA), are being assessed as non-invasive liquid biopsy tools for early detection [22,23,24,25,26,27]. Similarly, methylation-based biomarkers, such as hypermethylation of tumor suppressor genes, such as septin 9 (SEPTIN9), Ras association domain family member 1 (RASSF1A), adenomatous polyposis coli (APC), and cyclin dependent kinase inhibitor 2A (CDKN2A/p16), give way for early HCC detection through blood-based assays [28].

For prognostic markers, AFP remains a key player, as levels above 400 ng/mL are correlated with poor prognosis, increased tumor burden, and a higher risk of recurrence post-treatment [29]. High DCP levels demonstrate a correlation with poor survival outcomes and a stronger likelihood of vascular invasion [30]. Genetic and molecular alterations, such as TP53 mutations, have been frequently identified in aggressive HCC cases and are connected to poor prognosis, while CTNNB1 (β-catenin) mutations are coupled with better outcomes but also resistance to immune checkpoint inhibitors [31].

The tumor-immune microenvironment has an influential part in prognosis. High PD-L1 expression and elevated neutrophil-to-lymphocyte ratio (NLR) have been showcased as poor prognostic markers [32,33]. High transforming growth factor **β** (TGF-β) signaling activity is associated with immune evasion and tumor progression, while high VEGF expression anticipates a response to anti-angiogenic therapies such as sorafenib [34,35]. Overall, AFP, DCP, and AFP-L3 persist as the most commonly used diagnostic biomarkers for HCC, while genomic alterations (TP53), immune markers (PD-L1, NLR), and liquid biopsy-based markers (ctDNA, miRNAs) are growing in popularity as diagnostic/prognostic tools.

In this manuscript, we will analyze the functionality of Astrocyte elevated gene-1/Metadherin (AEG-1/MTDH) as an oncogene, pointing to factors that make it a viable therapeutic target for HCC. AEG-1 was first cloned as an HIV-1- and tumor necrosis factor α (TNFα)-inducible gene in primary human fetal astrocytes in 2002, with the subsequent identification of the localization of the protein in the endoplasmic reticulum (ER) in 2005 [36,37]. In parallel, in 2004, AEG-1 was cloned as a cell membrane protein facilitating breast cancer metastasis and was termed metadherin and given the GenBank symbol MTDH [38]. In the same year, two separate groups cloned the rodent version of the gene, coining the name lysine-rich CEACAM-1 co-isolated protein (LYRIC) and observing the presence of the protein in the ER, nuclear envelop, and tight junction [39,40]. Over the last 20 years, studies on AEG-1 have shown remarkable implications for contributing to the progression of all cancers studied, including HCC. AEG-1 overexpression is observed in many different cancers, including HCC, which is also linked to poor disease outcomes. AEG-1 plays a fundamental role in oncogenic transformation and angiogenesis, mechanisms that are vital for metastasis and tumor growth to occur [41]. Due to the key role AEG-1 plays in supporting tumorigenesis and its association with unfavorable medical outcomes, it presents as a promising focus for therapeutic intervention in HCC.

## 2. Regulation of Expression and Function and Structural Motifs in AEG-1

### 2.1. AEG-1 Gene and Its Regulation of Expression in Cancer

**Figure 1:**
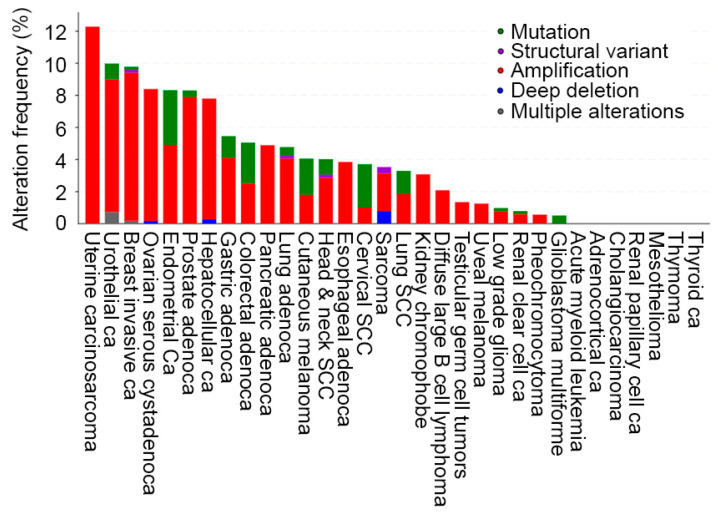
*AEG-1/MTDH* gene is amplified in human cancers. Analysis of alteration frequency of *AEG-1/MTDH* gene in the indicated cancers in The Cancer Genome Atlas (TCGA) PanCancer Atlas database. *AEG-1/MTDH* gene is amplified at variable frequency in a majority of the cancers. Figure created in cBioPortal (https://www.cbioportal.org/).

AEG-1 is encoded by the *MTDH* gene located on chromosome 8q22.1 [37]. The gene consist of 12 exons, and, due to alternative splicing, it is able to produce diverse transcript variants, although no correlation was observed between these variants and tumor stage and patient survival [37,42]. AEG-1 is ubiquitous in its expression in various tissues, with elevated levels detected in tissues containing muscular actin, such as the heart and skeletal muscle and tongue, as well as in the liver, adrenal gland, and thyroid [37]. Diverse mechanisms regulate its expression in cancer, such as chromosomal amplifications of 8q22, which show variability in different cancers (Figure 1). For example, breast cancer patients with poor clinical outcome showed gains of chromosome 8q22 that contained the AEG-1/MTDH gene [43]. Interestingly, while *AEG-1/MTDH* gene amplification was observed in HCC patients, no such gain was detected in cholangiocarcinoma patients, thereby stressing the importance of AEG-1 in HCC regulation (Figure 1) [44]. AEG-1 expression can be induced by oncogenes, such as Ha-ras and MYC [45]. It was documented that Ha-ras activates the phosphatidylinositol-3 kinase/AKT kinase (PI3K/AKT) pathway leading to the binding of MYC to AEG-1 promoter, thereby regulating its transcription [45]. Pro-inflammatory cytokines, such as interleukin-1β (IL1B) and TNFα, and lipopolysaccharide (LPS) induce AEG-1 expression via activating Nuclear factor-kappa B (NF-κB) thereby establishing a link between AEG-1 and chronic inflammatory cancers like HCC [46,47,48]. Multiple tumor-suppressor miRNAs, such as miR-30a-5p, miR-195, miR-302c, miR-375, and miR-497, target AEG-1 so that their downregulation in cancers results in the upregulation of AEG-1 in a post-transcriptional manner [49,50,51,52,53,54]. On the other hand, long non-coding RNAs (lncRNAs) can sponge AEG-1-targeting miRNAs, thereby upregulating AEG-1 [55,56,57,58]. Post-translationally, monoubiquitination causes the stabilization of AEG-1 protein in cancer cells, although the ubiquitin ligase mediating this effect is yet to be identified [59]. In glioblastoma cells, cytoplasmic polyadenylation element binding protein 1 (CPEB1) increases AEG-1 translation by binding to its mRNA, while in HCC cells CPEB3 inhibits AEG-1 translation by binding to the 3′-untranslated region of AEG-1 mRNA [60,61]. Thus, a plethora of mechanisms can drive the overexpression of AEG-1 in cancer cells, making it one of the most highly overexpressed oncogenes across all cancers.

### 2.2. AEG-1 Protein: Structure, Motifs, and Key Residues

**Figure 2:**
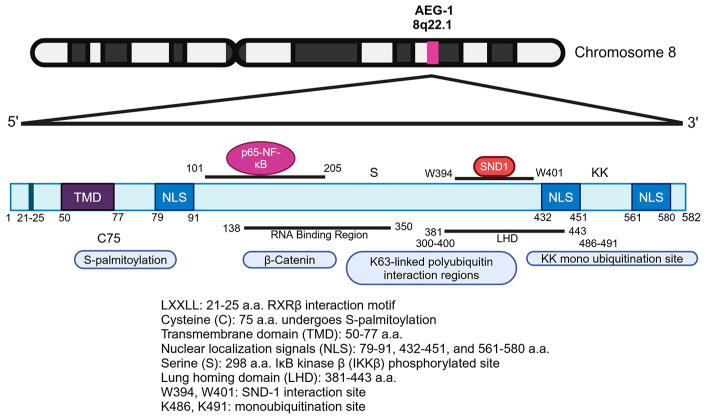
Important structural features and motifs of AEG-1. Human *AEG-1/MTDH* gene is located in chromosome 8q22.1, which codes for a 582 amino acid protein. AEG-1 harbors a transmembrane domain (TMD) and three nuclear localization signals (NLS). The detailed description and importance of different amino acid residues and interacting regions are provided in the text. Figure created in BioRender (https://www.biorender.com/).

AEG-1 is a highly basic protein which is rich in lysine residues and which contains 582 amino acids (a.a.) in humans (Figure 2) [37]. The protein is highly conserved in vertebrates and is not present in non-vertebrates, indicating that it evolved to perform specific functions in higher organisms [62]. Inspection of the amino acid sequence of AEG-1 does not identify any known domains or motifs that can classify it into a specific group and predict its function. The complete three-dimensional structure of the AEG-1 protein has not been resolved, and, as such, a precise understanding of its functional capacity cannot be ascertained. However, studies using diverse strategies have detected specific regions in the AEG-1 protein, providing clues on how it interacts and functions and indicating that AEG-1 exerts its functions by interacting with a variety of proteins and RNAs. Additionally, the subcellular localization of AEG-1 also helps explain its functions. Between 50 and 77 a.a. residues, the AEG-1 protein harbors a transmembrane domain (TMD), facilitating its anchoring into the ER membrane (Figure 2) [37,38,40,63,64]. The N-terminal 1–50 a.a. residues reside inside the ER lumen, while the remaining part of the protein resides in the cytoplasm [63]. In this location, AEG-1 functions as an RNA-binding protein (RBP) mainly regulating protein translation, and, although it lacks the consensus RNA-binding domains of RBPs, AEG-1 shares the structural property of high intrinsic disorder with other RBPs, and deletion mutation studies have mapped the AEG-1 RNA-binding region to the central disordered region between 138 and 350 a.a. [63]. NF-κB is a transcription factor that is sequestered in the cytoplasm by IκBα [65]. The classical NF-κB signaling pathway involves the binding of a ligand, such as tumor necrosis factor α (TNFα), to its cognate receptor, followed by the formation of multi-protein complexes that result in IκB kinase (IKK)-mediated phosphorylation and the eventual degradation of IκBα, facilitating the nuclear translocation of NF-κB so that it can regulate gene transcription [65]. The stimulation of NF-κB involves Lys63 (K63)-linked ubiquitylation of specific components of NF-κB signaling pathways, such as TNF receptor associated factor 2 (TRAF2) and receptor interacting serine/threonine kinase 1 (RIPK1/RIP1), via Lys63 (K63) linkage [64]. It was documented that ER-anchored AEG-1 directly interacted with the K63-linked ubiquitinated proteins via a.a. 300–400 residues, thereby serving a pivotal role in NF-κB activation [64]. The same TMD also allows AEG-1 to anchor into the cell membrane, especially in aggressive cancer cells, facilitating metastasis [38].

The basic lysine-rich regions in a.a. 79–91, 432–451, and 561–580 of the AEG-1 protein contain three nuclear localization signals (NLS) (Figure 2), and deletion analyses have identified that NLS1 and NLS3 and their flanking regions allow AEG-1 to localize to the nucleus and nucleolus [40,59]. In primary mouse hepatocytes, as well as in benign human lung, prostate, and thyroid tissues, AEG-1 is mainly localized in the nucleus, whereas, in cancer cells, it is predominantly located in the cytoplasm [59,66]. In the nucleus, AEG-1 is sumoylated and its subsequent monoubiquitination allows it to translocate to the cytoplasm with an increase in its half-life [59,66]. K486 and K491 residues in the extended NLS2 region of AEG-1 were shown to undergo monoubiquitination, and the E3 ubiquitin ligase TOPORS (topoisomerase I binding, arginine/serine-rich) was implicated to mediate this reaction although it was not functionally validated [67]. As yet, mutagenic studies have not been performed in in vivo settings to delineate the role of monoubiquitination in regulating AEG-1 function in normal physiology and in disease. While TNFα induces AEG-1 expression, it also induces the translocation of AEG-1 from the cytoplasm to the nucleus where it stimulates NF-κB activation [68,69]. However, the precise mechanism of AEG-1 shuttling between the cytoplasm and nucleus is unclear. How AEG-1 is released from being anchored in the ER or cell membrane and enters the nucleus or how it is exported out of the nucleus still remains to be studied.

AEG-1 harbors a lung homing domain (a.a. 381–443), which facilitates the adhesion of breast cancer cells to lung endothelium and which contributes to the metastasis of these cells to the lungs (Figure 2) [38]. Ligand-dependent transcription factors, known as nuclear receptors, contain an ‘LXXLL’ motif through which they interact with transcription co-activators and activate transcription [70]. AEG-1 does not have DNA-binding motifs, but it harbors an ‘LXXLL’ motif in 21–25 a.a. residues. A yeast two-hybrid (Y2H) assay using the N-terminal 57 a.a. of AEG-1 as bait identified Retinoid X receptor β (RXRβ) as an AEG-1-interacting protein, and mutagenesis studies unraveled that this interaction is mediated via the ‘LXXLL’ motif [66]. AEG-1 has only one cysteine in a.a. 75, which undergoes S-palmitoylation by zinc finger DHHC-type palmitoyltransferase 6 (ZDHHC6) [71]. The functional significance of RXR interaction and palmitoylation will be described in detail in later sections. IκB kinase β (IKKβ) phosphorylates AEG-1 at Serine 298, which is necessary for the subsequent phosphorylation and degradation of IκBα, nuclear translocation of NF-κB, and NF-κB-dependent gene expression [72]. Co-immunoprecipitation (co-IP) followed by mass spectrometry (mass spec) identified staphylococcal nuclease and Tudor domain containing 1 (SND1) as the strongest AEG-1 interacting protein, and this interaction has been shown to be a key mediator of AEG-1′s oncogenic effect [73,74]. W394 and W401 were identified as key residues of AEG-1 interacting with SND1, and the crystal structure of the AEG-1/SND1 interaction region was resolved, paving the way for developing small molecule inhibitors that perturb this interaction, inhibit AEG-1, and serve as anti-cancer agents [75,76,77,78].

## 3. AEG-1 Function in Normal Physiology

AEG-1 has been documented to play a major role in cancer progression; however, AEG-1 is also vital for maintaining physiological functions, including fertility, stress response, metabolism, and inflammation, which has been unraveled by analyzing AEG-1 knockout mice [79,80,81,82]. Mice deficient in AEG-1 (AEG-1^−/−^) are viable and do not exhibit overt developmental abnormalities, indicating that AEG-1 is not essential for organ development [81]. However, male AEG-1^−/−^ mice are infertile due to a significant loss of spermatozoa because of meiotic failure, highlighting AEG-1′s role in reproductive biology [79]. The impairment of spermatogenesis was related to unrepaired DNA damage and the altered expression of Piwi-interacting RNAs (piRNAs) and the decreased expression of miR-16 and miR-19b, which are known to be downregulated in the semen of infertile men [79]. These intriguing observations raise a plethora of questions, such as why this effect is male-specific, how AEG-1 regulates the expression of these small RNAs, and how these small RNAs regulate sperm fidelity. Unfortunately, no follow-up study was performed to address these questions. Although AEG-1 has been implicated in positively regulating glutamate transporter (excitatory amino acid transporter 2; EAAT2) expression in the context of glioma [83], no behavioral abnormality was observed in AEG-1^−/−^ mice suggesting that neurotransmitter regulation in the brain may not be a physiological function of AEG-1 [81].

Compared to WT mice, AEG-1^−/−^ mice were significantly leaner with less body fat and they also lived significantly longer [80]. When fed a high-fat diet (HFD), AEG-1^−/−^ mice did not gain body weight, whereas their WT littermates gained weight rapidly [80]. This effect was due to decreased fat absorption from the intestines, not because of an abnormality in fat consumption or fat synthesis [80]. As previously mentioned, AEG-1 uses the ‘LXXLL’ motif to interact with the transcription factor RXR, which prevents the recruitment of transcription co-activators, such as SRC-1, to RXR, thus preventing RXR-mediated gene transcription [66]. RXR serves as the obligate heterodimer partner of ligand-dependent nuclear receptors which regulate lipid metabolism, such as peroxisome-proliferator-activated receptor alpha and gamma (PPARA and PPARG, respectively), nuclear receptor subfamily 1 group H member 3 (NR1H3/LXR), and nuclear receptor subfamily 1 group H member 4 (NR1H4/FXR), nuclear receptors that regulate hormone and vitamin function, such as the thyroid hormone receptor (TR), vitamin D receptor (VDR) and vitamin A receptor (RAR), and those that regulate xenobiotic metabolism, such as nuclear receptor subfamily 1 group I member 2 (NR1I2/PXR) and nuclear receptor subfamily 1 group I member 3 (NR1I3/CAR) [84]. AEG-1 negatively regulates the function of all these transcription factors [85]. In AEG-1^−/−^, enterocytes’ expression of LXR target genes, which include cholesterol efflux pumps, was increased, thereby preventing cholesterol absorption [80]. PPARA is a master regulator of fatty acid β-oxidation (FAO) [86], and increased activity of PPARA in AEG-1^−/−^ enterocytes contributed to increased FAO and the decreased absorption of fatty acids into the circulation [80]. Cholesterol metabolites, such as oxysterols, and fatty acids serve as ligands for LXR and PPARA, respectively, and these compounds are enriched in HFD, further inhibiting fat absorption when fed HFD [80]. Caloric restriction increases longevity [85], and the AEG-1^−/−^ mice showed inhibition of aging-associated inflammation [81], the collective effect of which might contribute to longer survival in AEG-1^−/−^ mice.

In 16-month-old mice, the increased infiltration of macrophages was observed in AEG-1^−/−^ mice’s liver and spleen but not in their WT littermates, indicating that AEG-1^−/−^ mice are protected from aging-associated inflammation [81]. The underlying molecular mechanism lies in AEG-1′s ability to regulate the pro-inflammatory NF-κB signaling pathway by multiple mechanisms. Upon treatment with TNFα, AEG-1 translocates to the nucleus, where it directly interacts with the p65 subunit of NF-κB and CREB binding protein, (CREBBP/CBP) facilitating interaction between NF-κB and basal transcriptional machinery, promoting NF-κB-induced transcription [68,69]. On the ER membrane, AEG-1 facilitates the assembly of TRAF2, RIPK1, and other upstream regulators of NF-κB signaling [64], and IKKβ-mediated phosphorylation of AEG-1 allows I*κ*B*α* degradation and nuclear translocation of NF-κB [72]. Thus, AEG-1 is at multiple focal points of NF-κB activation. Indeed, in AEG-1^−/−^ hepatocytes and macrophages, LPS-induced NF-κB activation is robustly abrogated [81]. AEG-1^−/−^ mice were protected from dextran sodium sulfate (DSS)-induced colitis, further supporting the importance of the pro-inflammatory function of AEG-1 [87]. AEG-1 levels in macrophages were significantly higher than those in hepatocytes [82]. Employing a myeloid cell-specific conditional AEG-1 knockout mouse (AEG-1^ΔMAC^) and in vitro cultures of AEG-1^−/−^ macrophages, it was demonstrated that AEG-1 deficiency significantly abrogates macrophage function making the mice anergic [82]. This mouse model will be useful to further tease out the role of AEG-1 in macrophages in other inflammatory diseases conditions.

Compared to WT hepatocytes, AEG-1^−/−^ hepatocytes were more sensitive to oxidative and hypoxic stresses, as well as chemotherapy-induced cell death [81]. Additionally, these cells were more sensitive to hepatocarcinogen N-nitrosodiethylamine (DEN)-induced DNA damage and cell death [81]. In in vitro culture, WT hepatocytes start showing features of senescence after 4 days which was accelerated in AEG-1^−/−^ hepatocytes [81]. Conversely, senescence was markedly delayed upon the overexpression of AEG-1 in primary human hepatocytes or in hepatocytes obtained from hepatocyte-specific AEG-1 transgenic mice (Alb/AEG-1) [88]. Alb/AEG-1 hepatocytes also showed the increased activation of pro-survival pathways, such as extracellular signal-regulated kinase*/*mitogen-activated protein kinase 1 (ERK/MAPK1) and AKT, and increased expression of anti-apoptotic proteins of the B cell leukemia/lymphoma 2 (BCL2) family, which protected them from growth factor deprivation-induced cell death [88]. AEG-1 overexpression significantly protected from serum starvation-induced death in primary and immortal human fetal astrocytes, immortal melanocytes, and rat embryonic fibroblasts by activating the PI3K/AKT pathway [89]. Additionally, under serum-starved conditions, AEG-1 could provide survival benefits to immortal human fetal astrocytes by activating protective autophagy via a noncanonical pathway which involved autophagy related 5 (ATG5) upregulation [90]. Collectively, these findings identify AEG-1 as a protein that allows normal cells to withstand the detrimental effects of a variety of stressors. These observations showcase the importance of AEG-1 in many normal biological processes, as well as its contribution to ensuring cellular equilibrium across different organ systems and tissues.

## 4. Role of AEG-1 in Regulating MASH: A Precursor to HCC

**Figure 3:**
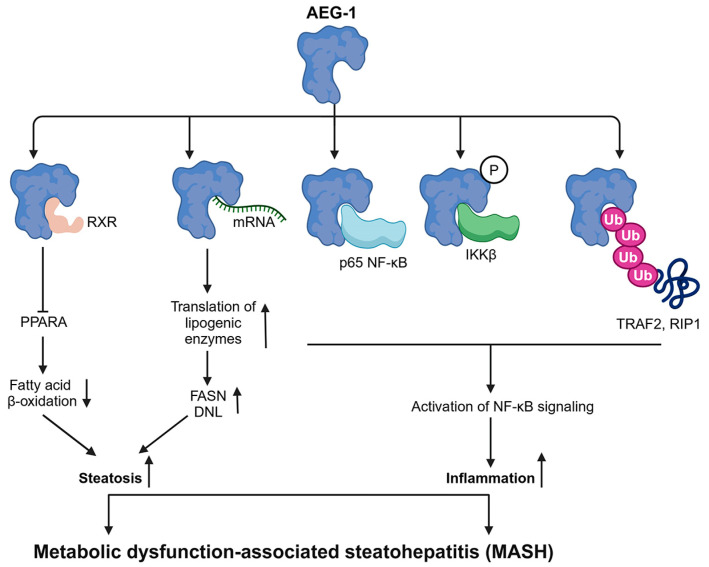
Mechanisms by which AEG-1 induces MASH. By using LXXLL motif, AEG-1 binds to RXR, thereby inhibiting PPARA and decreasing fatty acid β-oxidation. AEG-1 binds to mRNAs encoding lipogenic enzymes, such as fatty acid synthase (FASN), and increases their translation, resulting in increased de novo lipogenesis (DNL). Together, these events lead to steatosis. Multiple mechanisms activate NF-κB signaling pathway by AEG-1. AEG-1 binds to p65 subunit of NF-κB and functions as a bridge between NF-κB and basal transcription machinery. AEG-1 is phosphorylated by IKKβ facilitating nuclear translocation of NF-κB. AEG-1 interacts with upstream ubiquitinated molecules of NF-κB pathway, such as TRAF2 and RIP1. Activation of NF-κB leads to increased inflammation. Figure created in BioRender (https://www.biorender.com/).

MASH (previously known as non-alcoholic steatohepatitis or NASH)-induced chronic inflammation is the fastest-growing cause of HCC [8]. MASH has two major components: steatosis, in which lipids, predominantly triglycerides, accumulate in the liver, and inflammation [48,91]. AEG-1 directly augments both these components. A hepatocyte-specific AEG-1 transgenic mouse (Alb/AEG-1) spontaneously developed MASH and a hepatocyte-specific conditional AEG-1 knockout mouse (AEG-1^ΔHEP^) was protected from high fat diet (HFD)-induced MASH [48]. As described earlier, AEG-1 inhibits RXR function, and interestingly, in vivo, this inhibitory effect of AEG-1 is specifically skewed to PPARA [48]. PPARA-mediated fatty acid β-oxidation (FAO) was drastically inhibited in Alb/AEG-1 mice, thus causing steatosis, and was augmented in AEG-1^ΔHEP^ mice thus providing protection from developing HFD-induced steatosis [48]. As an RNA-binding protein anchored into the ER membrane [63], AEG-1 binds to fatty acid synthase (FASN) mRNA, facilitating its association with polysomes and increasing FASN translation [48]. An increase in FASN levels augments de novo lipogenesis (DNL), which also contributes to steatosis [48]. Besides FASN, AEG-1 bound to mRNAs encoding for fatty acid synthesizing enzymes and Gene Ontology (GO) analysis of AEG-1 bound mRNAs identified lipid metabolism-associated proteins as the most prominent category [63]. AEG-1 is an essential component of the activation of NF-*κ*B signaling [64,68,69,72], a master regulator of inflammation, thereby promoting the inflammatory aspect of MASH when AEG-1 is overexpressed [48]. AEG-1 expression is increased in fibrotic liver, it is induced in activated hepatic stellate cells (HSC) by TGF*β* and LPS, and the knockdown of AEG-1 inhibited HSC activation and induced apoptosis [92]. Thus, AEG-1 contributes to both inflammatory and fibrotic events associated with MASH (Figure 3).

The importance of the ‘LXXLL’ motif in regulating AEG-1 biology was interrogated using AEG-1-L24K/L25H mice in which the ‘LXXLL’ motif was mutated to ‘LXXKH’ using CRISPR/Cas9 technology [93]. As expected, the PPARA function was augmented in AEG-1-L24K/L25H mice, which provided partial protection from HFD-induced steatosis [93]. Unexpectedly, AEG-1-L24K/L25H livers presented with increased levels of lipogenic enzymes and increased mitogenesis and inflammation, suggesting that the inhibition of the AEG-1/RXR interaction in AEG-1-L24K/L25H mice allows AEG-1 to interact more with its other interacting partners, augmenting their activity [93]. For example, in AEG-1-L24K/L25H livers, the rate of AEG-1 binding to mRNAs for lipogenic enzymes was higher, facilitating their increased translation and DNL. AEG-1-induced NF-κB activity was increased, and oncogenic signaling pathways, such as ERK and AKT, were further activated [93]. These findings suggest that the ‘LXXLL’ motif maintains a balance among the pleiotropic functions of AEG-1, such that its loss protects from steatosis but augments the inflammatory and oncogenic functions of AEG-1.

S-palmitoylation, the addition of C16 palmitic acid to cysteine residues, is a major post-translational mechanism regulating protein structure, assembly, maturation, trafficking, and function [94,95]. It was documented that the only cysteine residue at a.a. 75 of AEG-1 is palmitoylated by ZDHHC6, and the mutation of Cys75 to serine (C75S) or alanine (C75A) abolishes the palmitoylation [71,96]. An AEG-1-C75S knock-in mouse was generated by CRISPR/Cas9, which did not show any developmental abnormality; neither did it show alterations in AEG-1 localization to ER [71]. However, RNA-sequencing (RNA-seq) analysis of isolated hepatocytes documented the activation of signaling pathways and upstream regulators that regulate cell proliferation, motility, inflammation, angiogenesis, and lipid accumulation in AEG-1-C75S mice, suggesting that palmitoylation keeps the oncogenic and MASH-promoting function of AEG-1 in check [71]. Indeed, our preliminary studies document increased steatosis in male and female AEG-1-C75S livers compared to AEG-1-WT, when fed a high-fat/high-sugar diet (unpublished data). In the liver, hepatocytes are organized into periportal, midlobular, and pericentral zones, and zone-specific dysregulation of gene expression perturbs metabolic activities contributing to MASH and, eventually, HCC [97]. Spatial transcriptomics (ST) analysis was performed on the livers of the AEG-1-WT and AEG-1-C75S littermates to identify how palmitoylation affects gene regulation by AEG-1 in different hepatic zones [98]. It was observed that the pathways promoting inflammation, MASH, and HCC were activated in the periportal and pericentral hepatocytes of AEG-1-C75S mice compared to AEG-1-WT mice [98]. However, in the midlobular zone, xenobiotic metabolism pathways, as well as PXR/RXR and LXR/RXR activation, were significantly inhibited in AEG-1-C75S mice versus AEG-1-WT, suggesting that AEG-1 exerts zone-specific functions in the liver, and palmitoylation modulates these functions differentially [98]. These findings also indicate the dominant positive function of AEG-1-C75S so that there is abrogation of RXR-mediated gene transcription. Another independent study further extended these observations [96]. It was documented that while ZDHHC6 palmitoylates AEG-1, palmitoyl-protein thioesterase 1/2 (PPT1/2) depalmitoylates it, thereby creating a dynamic balance [96]. Treatment with DEN augmented HCC development in AEG-1-C75A and ZDHHC6 knockout mice, and treatment with a PPT1 inhibitor hydroxychloroquine (HCQ) inhibited tumor growth in the s.c. xenograft model using HuH-7 cells [96]. AEG-1-C75A displayed increased AEG-1 protein stability, stronger interaction with SND1, and augmented RISC activity, resulting in the downregulation of tumor suppressor mRNAs [96]. Collectively, these studies suggest that boosting AEG-1 palmitoylation might be a potential strategy to protect from MASH and HCC. HCQ has been shown to enhance the anti-tumor activity of anti-PD-1 antibodies in melanoma [99]. The anti-PD-1 antibody nivolumab showed a durable objective response in advanced HCC patients [100], and a combination of the anti-PD-1 antibody and HCQ might be a potential treatment option for HCC.

## 5. Oncogenic Role of AEG-1

AEG-1 plays a crucial role in tumor development and progression. Overexpression of AEG-1 augments cancer hallmarks, resulting in the development of highly aggressive, angiogenic, chemoresistant, and metastatic cancers. AEG-1 is a scaffold protein, and it binds to both proteins and RNAs, resulting in a diverse array of effects. Here, we will focus on key events, mediated by AEG-1, that have been documented in multiple cancers, including HCC. We will highlight a few unique features of the AEG-1 function that have been shown only in particular cancers.

### 5.1. Interaction and Cooperation of AEG-1 with Other Proteins and RNAs

#### 5.1.1. Interaction with SND1: A Key Mechanism Mediating AEG-1′s Oncogenic Functions

Even though multiple studies have documented the tumor-promoting function of AEG-1 in a variety of cancers, the underlying mechanism was elusive, prompting the search for AEG-1-interacting proteins that can explain the biology. Screening of the human liver cDNA library by Y2H assay and co-IP/mass spec approaches has identified SND1 as the strongest AEG-1-interacting protein [73,74]. SND1 protein is composed of four staphylococcal nuclease (SN) domains, mediating DNA or RNA degradation, and a methyl-lysine and methyl-arginine-binding Tudor domain [101]. SND1 exerts a plethora of functions, including regulation of gene transcription, RNA splicing, gene silencing and RNA metabolism. SND1 is localized both in the nucleus and cytoplasm. In the nucleus, it functions as a transcription co-activator, e.g., as for signal transducer and activator of transcription 6 (STAT6), and also participates in mRNA splicing [102,103]. In the cytoplasm, SND1 is a component of RNA-induced silencing complex (RISC) facilitating gene silencing by small RNAs, such as siRNA or miRNA [104]. SND1 has also been shown to cleave hyper-edited double-stranded RNA (dsRNA) substrates generated by adenosine deaminases that act on RNA (ADARs) [105]. In HCC cells, the interaction of AEG-1 and SND1 was observed in the cytoplasm, and the role of both these proteins in maintaining optimum RISC activity was documented [73]. The overexpression of AEG-1 or SND1 augmented RISC activity with a resultant increased degradation of tumor-suppressor mRNAs targeted by oncogenic miRNAs, e.g., there was downregulation of PTEN which is a target of miR-221, an oncogene for HCC [73]. SND1 is overexpressed in many cancers, including HCC [73]. Overexpression and knockdown studies in human HCC cells and studies using a hepatocyte-specific SND1 transgenic mouse established SND1 as an oncogene for HCC, and a selective SND1 inhibitor, 3′,5′-deoxythymidine bisphosphate (pdTp) inhibited growth of human HCC tumors in s.c. or the orthotopic model in mice [73,106,107]. In breast cancer, AEG-1 has been shown to promote the expansion of tumor-initiating cells (TICs) facilitating metastasis, and AEG-1/SND1 interaction, followed by stabilization of SND1 protein, was shown to be a key event in this process [108]. SND1 levels were similar in WT and AEG-1 knocked-down breast cancer cells under steady-state conditions [108]. DNA replication stress is observed during tumor initiation, and the induction of such stress in AEG-1 knocked-down cells significantly reduced the half-life of SND1 protein, and, conversely, AEG-1 overexpression increased SND1 protein stabilization upon heat shock, suggesting that AEG-1/SND1 interaction aids in cell survival under stressful conditions [75,108]. Indeed, a lack of tumorigenic potential was demonstrated in AEG-1 mutants that did not interact with SND1 [75,108]. Studies have also highlighted the relevance of AEG-1/SND1 interaction in metastatic clear cell renal cell carcinoma [109]. In prostate cancer cells, the AEG-1/SND1 complex recruited the oncogenic ETS-domain transcription factor ERG via the Tudor domain of SND1 [110]. ERG promoted nuclear localization of AEG-1/SND1, which promoted cell proliferation and the deletion of SND1-reduced tumor burden in an ERG-overexpressing mouse prostate cancer model [110]. The critical importance of AEG-1/SND1 interaction spurred the development of strategies to inhibit the interaction, which will be described in detail in a later section.

#### 5.1.2. Cooperation of AEG-1 and MYC

**Figure 4:**
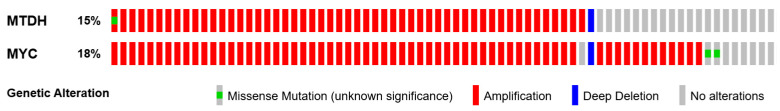
Co-amplification of *AEG-1/MTDH* and *MYC* genes in human HCC in The Cancer Genome Atlas (TCGA) (*n* = 379). Each bar represents one patient, and a fraction of the total 379 patients, which shows amplification of *AEG-1/MTDH* and *MYC*, is displayed in the figure. Fifteen percent of HCC patients show co-amplification of *AEG-1* and *MYC* genes. Figure created in cBioPortal (https://www.cbioportal.org/).

MYC, encoding c-Myc, is a driver oncogene for HCC [111]. MYC directly binds to the AEG-1 promoter and regulates its transcription [45]. AEG-1 interacts with and inhibits zinc finger and BTB domain containing 16 (ZBTB16/PLZF), a transcription repressor, which induces MYC transcription [112]. MYC is transcriptionally regulated by *β*-catenin, and AEG-1 induces MYC expression by activating the Wnt/*β*-catenin pathway in HCC cells [44]. MYC and AEG-1/MTDH genes are located in human chromosome 8q and these genes are co-amplified in ~15% of human HCC cases in the TCGA database (Figure 4). The overexpression of both AEG-1 and MYC in mouse hepatocytes (Alb/AEG-1/c-Myc mouse) resulted in the spontaneous development of metastatic HCC, which was further augmented upon DEN treatment [113]. The tumor burden in these mice was significantly higher than those expressing either gene alone (Alb/AEG-1 or Alb/c-Myc), and the latter did not develop metastasis [113]. Alb/AEG-1/c-Myc hepatocytes showed higher levels of proliferation, invasion, and chemoresistance compared to Alb/AEG-1 or Alb/c-Myc hepatocytes [113]. RNA-seq unraveled a unique gene signature characterized by the overexpression of non-coding RNAs (ncRNAs), such as Rian, Mirg, and Meg3, only upon the overexpression of both AEG-1 and MYC, and the knockdown of these ncRNAs abrogated proliferation and invasion in Alb/AEG-1/c-Myc hepatocytes [113]. These findings, unraveling cooperation between AEG-1 and MYC in generating aggressive HCC, pave the way for evaluating a combinatorial treatment strategy targeting these two proteins for HCC, which we are now actively pursuing.

#### 5.1.3. Implication of AEG-1/RXR Interaction for HCC

The interaction of AEG-1/RXR exerts multiple effects on HCC. In the nucleus, AEG-1 interacts with RXR and inhibits its function [66]. In HCC cells and Alb/AEG-1 hepatocytes, where AEG-1 is abundant in the cytoplasm, AEG-1 traps RXR in the cytoplasm and prevents its nuclear translocation [66]. AEG-1 also induces RXR phosphorylation, which inactivates RXR, via activating ERK [66]. Thus, in HCC cells, there are multiple mechanisms by which AEG-1 inhibits RXR function. Retinoic acid is known for its anti-cancer properties [114] and its receptor RAR regulates gene transcription by interacting with RXR [66]. Indeed, AEG-1 overexpression protected human HCC cells from killing by retinoids, such as all-trans retinoic acid (ATRA) and 9-cis retinoic acid, and the knockdown of AEG-1 potentiated this killing both in vitro and in vivo [66]. This strategy could be extended to acute myelogenous leukemia (AML), in which retinoids are used clinically as anti-cancer agents.

Various cancers are correlated with non-thyroidal illness syndrome (NTIS), characterized by low serum 3,5,3′-triiodothyronine (T3) and normal l-thyroxine (T4) levels [115]. The decreased activity of type I 5′-deiodinase (DIO1), which converts T4 to T3, is a pertinent contributor to the development of NTIS [115]. The binding of T3 to TR/RXR regulates gene expression, and DIO1 itself is a target of TR/RXR [116]. AEG-1 inhibited the TR/RXR function and, therefore, it downregulated DIO1 expression, and AEG-1 knockdown resulted in DIO1 upregulation [116]. In human HCC patients, an inverse correlation was observed between AEG-1 and DIO1 levels, and the sera of HCC patients and Alb/AEG-1 mice displayed low T3 and normal T4 levels [116]. These findings suggest that the overexpression of AEG-1 might be one mechanism of NTIS associated with HCC and other malignancies.

#### 5.1.4. Interaction of AEG-1 with RNAs

Several screening studies have identified AEG-1 as an ER mRNA-binding protein [117,118,119,120]. Functional studies utilizing high-throughput sequencing of RNA isolated by crosslinking immunoprecipitation (HITS-CLIP) and photoactivatable ribonucleoside-enhanced crosslinking and immunoprecipitation PAR-CLIP methods have unraveled AEG-1 RNA interactome, which is rich in transcripts encoding ER-resident and integral membrane proteins, as well as secretory and cytosolic proteins which function in mRNA localization, translation regulation, and RNA quality control [63]. These observations explain previous findings that AEG-1 binds to the mRNAs encoding a number of endomembrane or secreted proteins and facilitates their association with polysomes, leading to increased translation. These include multidrug resistance gene 1 (MDR1/ABCB1), conferring chemoresistance; coagulation factor XII (F12), facilitating angiogenesis; and FASN, promoting DNL and MASH [48,88,121]. Chemoresistance and angiogenesis are classical hallmarks of cancer, and MASH is an important determinant of HCC, indicating that the RNA-binding function of AEG-1 is a crucial event in hepatocarcinogenesis.

In medulloblastoma, lncRNA Miat/AEG-1 interaction helps maintain cancer stem cells by regulating the biogenesis of oncogenic miRNAs [122]. However, whether this observation can be generalized to other cancers, including HCC, remains to be determined.

#### 5.1.5. Additional AEG-1-Interacting Proteins Relevant to HCC

Recent co-IP/mass spec studies have identified novel AEG-1-interacting proteins in HCC which, although well-characterized, need to be validated by additional independent studies. DEAD-box helicase 17 (DDX17) was identified as an AEG-1-interacting protein in the nucleus of human HCC cells [123]. Deletion analysis revealed that the N-terminal 1-291 of AEG-1 and C-terminal helicase domain (405–553 a.a.) of DDX17 mediated the interaction, which enhanced the stability of the DDX17 protein [123]. DDX17 is a putative RNA helicase, but, in this context, it interacted with the transcription factor Y-box binding protein 1 (YB1) which, in turn, bound to the promoter of epidermal growth factor receptor (EGFR), inducing its transcription [123]. The resultant increase in EGFR augmented the oncogenic MEK/ERK pathway [123]. DDX17 overexpression was shown in human HCC, and studies using Ddx17 knockdown human HCC cells and hepatocyte-specific conditional DDX17 knockout mice unraveled an oncogenic role of DDX17 in promoting HCC [123].

Zinc finger and BTB domain containing 17 (ZBTB17), also known as Miz1, is a transcription factor that was shown to suppress HCC independent of its transcriptional activity [124]. Hepatocyte-specific conditional Miz1 knockout mice were more susceptible to DEN- and MASH-induced HCC, and single-cell RNA-sequencing (scRNA-seq) of the livers of these mice identified a subset of hepatocytes with hyperactivation of NF-*κ*B, which facilitated the polarization of tumor-infiltrating macrophages toward the pro-inflammatory M1 phenotype, thereby promoting liver inflammation [124]. AEG-1 was identified as a Miz1-interacting protein, and Miz1 inhibited AEG-1/RelA (p65 NF-κB) interaction as well as IKK-mediated AEG-1 phosphorylation. Thus, Miz1 mediated its tumor suppressor effect by preventing AEG-1-induced NF-*κ*B activation [124]. In human HCC patients, Miz1 levels inversely correlated with disease recurrence and poor prognosis, thus providing human relevance of the findings from the mouse models [124].

Protein arginine methyltransferase 5 (PRMT5) promoted proliferation and motility of HCC cells and was shown to interact with AEG-1 [125]. It was shown that AEG-1 interacts with β-catenin and keeps it in the cytoplasm [125]. PRMT5 overexpression competes with β-catenin and PRMT5/AEG-1 interaction frees β-catenin, allowing it to translocate to the nucleus and promote gene expression and HCC [125]. Although this is an intriguing observation, these studies need to be more in depth, using in vivo models to validate the findings. More importantly, it was shown that PRMT5 interacts with AEG-1 via AEG-1′s TMD, and it is not clear how a membrane-bound region of AEG-1 can interact with PRMT5.

A list of important proteins and RNAs interacting with AEG-1 in HCC cells is provided in Table 1.

### 5.2. Signaling Pathways Activated by AEG-1

**Figure 5:**
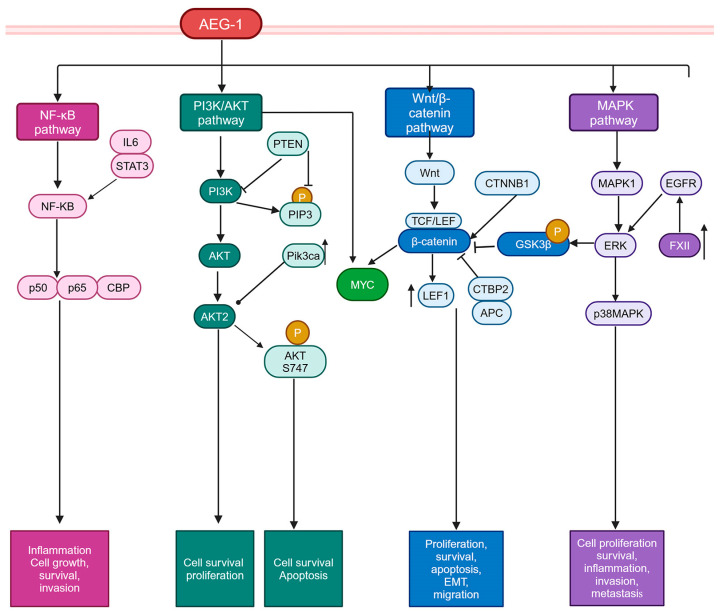
Signaling pathways activated by AEG-1 in HCC. AEG-1-induced activation of NF-κB contributes predominantly to inflammation. Activation of PI3K/AKT, Wnt/β-catenin, and MAPK pathways promote tumorigenesis by augmenting cell survival and proliferation, resistance to apoptosis, invasion, EMT, and metastasis. Figure created in BioRender (https://www.biorender.com/).

AEG-1 overexpression activates some of the key signaling pathways in cancer cells. Here, we predominantly focus on those pathways that are relevant to HCC (Figure 5).

#### 5.2.1. PI3K/AKT Pathway

The PI3K/AKT pathway is a major signaling pathway that regulates cell survival and proliferation, and activation of this pathway plays an essential role in hepatocarcinogenesis [126]. The PI3K/AKT pathway is negatively regulated by phosphatase and tensin homolog (PTEN), which dephosphorylates phosphatidylinositol 3,4,5-triphosphate (PIP3) generated by PI3K [127]. In ~40% of HCC patients, PTEN is inactivated by gene deletion or loss-of-function mutations, leading to the activation of the PI3K/AKT pathway [127]. The deletion of Pten from hepatocytes results in MASH and, subsequently, HCC [128]. On the other hand, phosphatidylinositol-4,5-bisphosphate 3-kinase catalytic subunit alpha (Pik3ca) overexpression in mouse hepatocytes generates steatosis and HCC, while deletion of Akt2 from mouse hepatocytes leads to the accumulation of triglycerides (TG) in the liver. PI3K/AKT pathway activation induces AEG-1 [129,130]. Activation of the PI3K/AKT pathway augments the activity of MYC [131], which might, in turn, induce AEG-1, although this hypothesis needs to be experimentally validated. AEG-1 activates the PI3K/AKT pathway, potentially through its interaction with AKT2, resulting in prolonged stabilization of phosphorylated AKT S747, which augments downstream signaling, including phosphorylation of AKT substrates, which regulate cell survival and apoptosis [132]. This phenomenon has been shown only in glioma cells [132] and needs to be validated in other cancers. Nevertheless, a key outcome of AEG-1-mediated PI3K/AKT activation is protection from serum starvation-induced apoptosis and anoikis in a variety of cancers, including HCC, as well as in Alb/AEG-1 hepatocytes [45,88,89,133]. Interestingly, both WT and AEG-1^−/−^ hepatocytes responded equally to EGF-induced activation of AKT and ERK [81], suggesting that activation of these pathways by AEG-1 might be selective to its oncogenic function rather than its normal physiological function.

#### 5.2.2. Wnt/β-Catenin Pathway

The Wnt/β-catenin pathway is essential for hepatobiliary development, and, in a healthy adult liver, it is primarily inactive but is re-activated in a variety of conditions, including HCC [134]. In the canonical pathway, the binding of the Wnt ligand to its receptor triggers the nuclear translocation of *β*-catenin, where it binds to T cell factor*/*lymphoid enhancer binding factor (TCF/LEF) transcription factors to regulate the transcription of oncogenic genes, including MYC [134]. Activating mutation in the *CTNNB1* gene, encoding β-catenin, is observed in ~30% of HCC cases [135]. In a search for the mechanism of the oncogenic function of AEG-1, global gene expression changes between the control, and AEG-1-overexpressed cells were analyzed, which unraveled alterations in genes in Wnt/β-catenin pathway upon AEG-1 overexpression [44]. AEG-1 overexpression induced LEF1, and its target gene MYC, expression. AEG-1 downregulated negative regulators of Wnt/β-catenin pathway, such as C-terminal binding protein 2 (CTBP2) and adenomatous polyposis coli (APC), and AEG-1-mediated activation of ERK resulted in the phosphorylation and degradation of glycogen synthase kinase β (GSK3β) which freed up β-catenin, allowing its nuclear translocation [44]. The knockdown of LEF1 significantly inhibited AEG-1-induced HCC cell proliferation and invasion. As described earlier, a role of PRMT5 is implicated in AEG-1-induced β-catenin activation in HCC cells [125]. Under steady-state conditions, no difference in Wnt/β-catenin pathway activation was observed between WT and AEG-1^−/−^ hepatocytes, suggesting that Wnt/β-catenin pathway activation is an oncogenic function of AEG-1 [81]. The Wnt/β-catenin pathway is required for embryogenesis and hepatobiliary development [134], and no abnormality in organ development was observed in AEG-1^−/−^ mice [81], further providing evidence for the distinction between physiological and oncogenic functions of AEG-1.

#### 5.2.3. MAPK Pathway

AEG-1 overexpression markedly activates the ERK/MAPK1 and p38 MAPK signaling pathways, and the inhibition of these pathways in human HCC cells markedly abrogated cell proliferation [44]. In Alb/AEG-1, hepatocytes sustained activation of EGFR, which accounted for the increased activation of ERK/MAPK1 [88]. Interestingly, proteomic analysis of conditioned media (CM) identified higher levels of several coagulation factors, notably FXII, in Alb/AEG-1 hepatocytes, which induced EGFR and subsequent ERK activation [88]. Thus, growth factor overexpression might be one mechanism by which AEG-1 activates the oncogenic ERK pathway.5.2.4. NF-κB pathway.

Physiologically, AEG-1 functions as a key regulator of inflammation by activating NF-κB [81]. This pathway is highly relevant for cancers arising from chronic inflammation, such as HCC [136]. AEG-1^−/−^ mice showed profound resistance to DEN-induced HCC, which was due to the inhibition of the NF-κB-dependent activation of oncogenic interleukin-6/signal transducer and the activator of the transcription 3 (IL6/STAT3) signaling pathway [81]. Macrophages play a central role in hepatocarcinogenesis by sustaining inflammation, and NF-κB activation in both hepatocytes and macrophages is necessary for HCC development [137,138]. DEN-induced HCC was partially attenuated in AEG-1^ΔHEP^ mice but completely abrogated in AEG-1^ΔMAC^ mice, further stressing the importance of AEG-1-induced inflammation in HCC [82]. AEG-1-mediated inflammation has been implicated in playing a role in other inflammatory cancers, such as gastric cancers, as well as pre-cancerous inflammatory conditions, such as MASH, and inflammatory diseases, such as rheumatoid arthritis, HIV-1-induced neuroinflammation, and diabetic kidney disease [46,48,139,140,141].

## 6. Clinical Correlation, and Diagnostic and Prognostic Values of AEG-1 in HCC

The first publication interrogating the role of AEG-1 in HCC checked the expression levels of AEG-1 by tissue microarray (TMA), containing 86 primary and 23 metastatic HCC and 9 adjacent normal liver samples, using immunohistochemistry [44]. A progressively rising AEG-1 staining was observed from stages I to IV and from well-differentiated to poorly differentiated HCC, with positive staining detected in ~93.6% of samples [44]. Very little to no staining was detected in adjacent normal liver tissue. AEG-1 mRNA levels were analyzed from Affymetrix microarray data from a normal liver (n = 10), cirrhosis (n = 13), low- and high-grade dysplasia (n = 10 and n = 8, respectively), and HCC (n = 91) conditions [44]. Compared to the normal liver and cirrhosis data, significant increases in AEG-1 mRNA levels were observed in dysplastic and HCC patients [44]. In 323 HCC patients, IHC analysis identified positive AEG-1 expression in 54.2% of patients [142]. In this study, AEG-1 levels did not correlate with age, gender, liver cirrhosis, serum α-fetoprotein, tumor diameter, tumor encapsulation, or Barcelona Clinic Liver Cancer (BCLC) stage [142]. However, a significant association between AEG-1 levels and microvascular invasion (*p* < 0.001), pathologic satellites (*p* = 0.007), tumor differentiation (*p* = 0.002), and TNM stage (*p* = 0.001) was observed [142]. Univariate and multivariate analyses identified AEG-1 as an independent prognostic factor for overall survival (HR = 1.870, *p* < 0.001) and recurrence (HR = 1.695, *p* < 0.001) [142]. These findings were corroborated in additional studies using independent HCC patient cohorts establishing AEG-1 levels as a prognostic marker for HCC patients [143,144,145].

AEG-1 and GPC3 levels were evaluated as potential diagnostic markers by IHC in HCC patient samples [146]. In HCC patients, AEG-1 and GPC-3 levels showed 92% and 54% positivity, respectively, compared to adjacent liver and dysplastic nodules [146]. While on its own, AEG-1 showed high sensitivity and low specificity and accuracy, and GPC3 showed high specificity but low sensitivity and accuracy; together, sensitivity, specificity, and accuracy reached 94.6%, 89.5%, and 90.5%, respectively, suggesting that this combination might be used as diagnostic markers for biopsy samples in HCC [146].

Because overexpressed AEG-1 is detected on the surface of cancer cells, it was hypothesized that an autoantibody against AEG-1 might develop in advanced cancer patients, which might be used as a disease marker. Enzyme-linked immunosorbent assay (ELISA) was performed using sera from 230 normal individuals and 483 different cancer patients, including 96 HCC patients, and AEG-1 lung homing domain (381–443 a.a.) was used as an antigen, to detect the auto anti-AEG-1 antibody [147]. While none of the normal individuals tested positive, the anti-AEG-1 antibody, at titers of >1:50, was detected in ~50% of all cancer patients, including HCC patients [147]. This study needs to be repeated, and the utility of this approach needs to be compared with AFP and other serum diagnostic markers for advanced HCC. Additionally, it needs to be checked whether anti-AEG-1 antibody could be a diagnostic marker for early cancers, for which specific biomarkers with clinical utility are still in dire need.

## 7. Therapeutic Targeting of AEG-1

Given its central role in cancer progression, multiple AEG-1-inhibitory strategies are being evaluated for their anti-cancer efficacy. Here, we highlight some of these appoaches with special emphasis on those which have been tested in vivo as well as those which are relevant to HCC (Table 2).

### 7.1. Gene Silencing Strategies for Targeting AEG-1

Gene silencing approaches have been extensively explored as a therapeutic strategy in liver diseases including HCC [150,151]. A number of gene silencing strategies that target AEG-1 expression, such as using small interfering RNA (siRNA) or antisense oligonucleotides (ASOs) have emerged as promising therapeutic approaches. These methods work by directly reducing the expression of AEG-1, thereby disrupting the downstream oncogenic signaling cascades that contribute to tumor development and progression. Targeted nanoparticles were developed to deliver AEG-1 siRNA specifically to hepatocytes [148]. These nanoparticles are based on positively charged polyamidoamine (PAMAM) dendrimers, which bind to negatively charged siRNA and polyethylene glycol (PEG), which increases circulation half-life and reduces charge density, hence toxicity, and galactose lactobionic acid (Gal), which specifically binds to asialoglycoprotein receptors expressed by hepatocytes, thereby facilitating targeting [148]. PAMAM-PEG-Gal-delivered AEG-1 siRNA on its own could significantly inhibit the growth of human HCC cell-derived orthotopic xenografts in nude mice, and, in combination with ATRA, completely inhibited the xenograft growth [148]. This approach also prevented the development of HFD-induced MASH in C57BL/6 mice, suggesting that targeted nanoparticle-delivered AEG-1 siRNA could be both a preventive and therapeutic approach for HCC [48].

An siRNA library screening approach identified AEG-1 as a regulator of PD-L1 expression, and it was demonstrated that the AEG-1-LEF1-β-catenin complex binds to PD-L1 promoter to regulate its transcription [149]. Subcutaneous tumors using Hepa1-6 cells were established in syngenic mice, and the mice were intraperitoneally injected with anti-PD-1 antibody and locked nucleic acid-modified (LNA) antisense oligonucleotide (ASO) for AEG-1 [149]. The combination showed significant inhibition of tumor growth and improved survival, compared to each agent alone, with increased infiltration of cytotoxic T cells in the tumor [149]. LNA ASO for AEG-1 also inhibited MC-38 and LLC1 tumors, representing colorectal and lung cancer, respectively, in immunocompetent mice, thereby establishing the utility of this approach in diverse cancers [152].

### 7.2. Inhibition of AEG-1/SND1 Interaction

Interaction with SND1 is a crucial event in mediating AEG-1 function, and resolution of the crystal structure of AEG-1/SND1 interaction domains [75] facilitated the development of approaches to inhibit this interaction. A small peptide, derived from 393 to 403 a.a. of AEG-1, was shown to inhibit AEG-1/SND1 interaction, and its ability to inhibit in vitro proliferation of triple-negative breast cancer cells was demonstrated [153]. However, this peptide was not tested in vivo. A small molecule compound library screening identified a number of compounds inhibiting AEG-1/SND1 interaction, of which the prototype molecule C26-A6 inhibited tumor growth and metastasis and enhanced sensitivity to paclitaxel and anti-PD-1 antibody in in vivo models of triple-negative breast cancer using 4TO7 cells and Balb/c mice [76,77]. C26-A6 treatment did not exert any toxicity, further stressing its therapeutic use [77]. C26-A6 works at μM concentrations in vitro, and a new compound C19 was generated which disrupted AEG-1/SND1 interaction at nM concentrations and also inhibited MCF-7 human breast cancer xenografts in nude mice [78]. These compounds have not been tested in other cancers, including HCC, and, as such, although promising, their utility requires further validation.

### 7.3. Synergy with Existing Therapies in HCC

AEG-1 is known to contribute to chemoresistance in various cancers [154]. In HCC, this effect is mediated in multiple ways. AEG-1 binds to ABCB1/MDR1 mRNA, increasing its translation, thereby promoting the efflux of drugs, such as doxorubicin (Dox) [63,121]. AEG-1 upregulates the transcription factor late SV40 factor (LSF/TFCP2), which regulates thymidylate synthase (TS), the substrate on which 5-fluorouracil (5-FU) works [155]. In addition, AEG-1 induces the 5-FU catabolizing enzyme dihydropyrimidine dehydrogenase (DPYD) which, in combination with increases in TS, confers resistance to 5-FU [155]. In xenograft experiments, a combination of AEG-1 shRNA with Dox or 5-FU profoundly inhibited HCC tumor growth [121,155]. A major target of miR-375 is AEG-1 [52]. Liposomes co-delivering miR-375 and Dox significantly inhibited xenografts of SMMC-7721 HCC cells, compared to each agent alone, without exerting discernable toxicity [156]. AEG-1 inhibition synergized with immunotherapy, such as anti-PD-1 antibody in HCC and other cancers [76,149].

Apart from those approaches, it was demonstrated that the adenovirus-mediated delivery of anti-AEG-1 single chain antibody fragment (ScFv), driven under the cancer-specific stathmin promoter, induced apoptosis in cervical cancer cells and inhibited xenografts of HeLa cells [157]. This promising approach needs further exploration in other cancer types.

### 7.4. Challenges in AEG-1-Targeting Therapy and Potential Solutions

One potential challenge in targeting AEG-1 is its wide-spread expression in various tissues, which raises concerns about off-target effects and toxicity. siRNAs designed to silence AEG-1 may inadvertently downregulate other genes with partial sequence complementarity, leading to unintended biological effects. AEG-1 does not have any homolog, nor does it have any major domains or motifs clustering it to a specific class of proteins, thereby potentially decreasing the likelihood of off-target effects. However, the saturation of the RISC by exogenous AEG-1 siRNA can disrupt endogenous microRNA pathways, thereby subscribing to off-target effects. siRNAs can trigger immune sensors, such as toll-like receptors (TLRs), leading to the production of cytokines and other inflammatory mediators, further complicating the effects [158]. To enhance the specificity of AEG-1-targeted therapies and reduce off-target effects, several strategic methods can be employed. The chemical modifications of siRNAs, such as the substitution of 2′ position of ribose with 2′-O-methyl, 2′-fluoro, 2′-deoxy, or locked nucleic acid, have been shown to reduce off-target interactions and immune activation [159]. These modifications can improve the stability of siRNAs and decrease their recognition by immune sensors. Another approach involves the use of advanced gene-editing technologies like CRISPR/Cas9. By formulating guide RNAs that specifically target the AEG-1 gene, it is possible to achieve precise gene knockouts or modifications. However, CRISPR/Cas9 is also subject to off-target effects, e.g., the Cas9 nuclease may introduce double-strand breaks at unplanned genomic locations [160]. Ongoing research aims to improve the specificity of CRISPR/Cas9 through the development of high-fidelity Cas9 variants and optimized guide RNA designs [161].

Apart from male infertility, AEG-1^−/−^ mice do not show any developmental abnormality [79,81], and their leanness has been attributed predominantly to an abnormality in fat absorption from the intestines [80], suggesting that AEG-1 inhibition may not have any significant adverse effect on normal cells, especially when used to treat cancers in adult individuals. This premise is supported by the lack of toxicity shown by AEG-1 siRNA, AEG-1 ASO, and small molecule inhibitors of AEG-1 in mice [77,78,148,149]. Nevertheless, as these strategies move from pre-clinical models to clinical trials, toxicity and off-target effects should be meticulously monitored. AEG-1 regulates macrophage function [82], inhibits antigen presentation, and dampens T cell function and T cell-mediated immunity [76]. AEG-1 inhibition, therefore, might make one susceptible to infection, especially in cancer patients whose immune function might already be compromised.

The intracellular localization of AEG-1 creates a compelling need for delivery systems capable of crossing cellular membranes to reach the cytoplasm or nucleus. Traditional delivery methods, such as viral vectors, pose risks related to immunogenicity and insertional mutagenesis, highlighting the need for alternative approaches [162]. Nanotechnology offers innovative solutions for the targeted delivery of therapeutic agents, enhancing specificity and reducing systemic toxicity [163]. Nanocarriers, including liposomes, nanoparticles, micelles, dendrimers, and nanogels, can be engineered to encapsulate drugs, nucleic acids, or proteins, protecting them from degradation and facilitating controlled release [163]. These systems can be modified with targeting ligands to recognize and bind to specific receptors overexpressed on cancer cells, thereby increasing the accumulation of therapeutics at the tumor site, thereby enhancing the therapeutic efficacy of the payload while minimizing adverse effects on normal tissues [163]. HCC develops on a liver injured by cirrhosis with compromised function. The detoxification of drugs is severely impaired in such a liver, and drug-induced toxicity is a major issue for HCC patients when completing a course of treatment. Gene-based therapies offer an alternative non-toxic approach [164]. The successful application of targeted nanoparticles to deliver AEG-1 siRNA to protect from MASH and HCC paves the way for further evaluation of the strategy for clinical trials [48,148].

## 8. Current Challenges and Knowledge Gaps

AEG-1 has garnered significant attention in oncology due to its overexpression in various cancers and its role in promoting tumor progression, metastasis, and chemoresistance. However, many unanswered questions remain for in-depth understanding of structural and functional aspects of AEG-1. The *AEG-1/MTDH* gene is amplified in HCC but not in cholangiocarcinoma (Figure 1). This finding raises the question of whether there is differential expression and function of AEG-1 in hepatocytes and cholangiocytes. If cell type-specific differential function of AEG-1 exists, it would be intriguing to study AEG-1′s role in regulating metabolism in these cell types, e.g., how AEG-1 regulates bile acid synthesis and metabolism. NR1H4/FXR is a major transcription factor which heterodimerizes with RXR and regulates bile acid metabolism [165]. AEG-1 inhibits RXR function [66], and it is anticipated that AEG-1 would modulate FXR function and, hence, bile acid metabolism, which needs to be experimentally explored. FXR functions as a tumor suppressor for HCC [166] and AEG-1-mediated inhibition of FXR might also contribute to hepatocarcinogenesis. FXR agonists are being evaluated for the treatment of MASH [167] and AEG-1 inhibition provides protection from HFD-induced MASH [48]. The crosstalk between AEG-1 and FXR in the liver is an important concept that remains to be interrogated.

AEG-1 is localized in ER and cell membranes as well as in the nucleus and nucleolus. However, the mechanism of AEG-1 trafficking and the determinants of its sub-cellular localization are still unclear. Cell-membrane-localized AEG-1 has been shown to promote metastasis [38]. The underlying mechanism, e.g., which extracellular matrix proteins it interacts with to mediate this effect, needs further interrogation. AEG-1 has been shown to interact with nucleolin in breast cancer cells [168], and nucleolin was also identified as an AEG-1-interacting protein in co-IP/mass spec analysis [73]. Elucidation of the role of AEG-1 in regulating nucleolar function requires more in-depth studies.

AEG-1 functions as an RNA-binding protein facilitating translation [63]. It is not clear what determines the specificity of AEG-1′s RNA binding. How AEG-1 selects the mRNAs encoding endomembrane proteins remains to be determined. As a scaffold protein, AEG-1 interacts with a wide array of proteins and protein complexes, and these interactions vary between normal and cancer cells. AEG-1 is not involved in the steady-state regulation of cell survival pathways [81]. However, it is vital for survival under stressful conditions [82,89]. AEG-1 depletion resulted in stress granule formation [169] but the importance of this observation needs further exploration. AEG-1 interacts with RXR but, in vivo, this inhibitory effect is skewed towards RXR heterodimer partner PPARA [48,66]. It would be interesting to check whether AEG-1 preferentially competes with PPARA co-activators, such as PPARG coactivator 1 alpha (PPARGC1A/PGC1α), and elucidate the mechanism of this preference. AEG-1 is required for the activation of macrophages and hepatic stellate cells [92]. Its function in tumor microenvironment is an understudied field that can be exploited for therapeutic intervention. These unexplored concepts and questions pave the way for multiple avenues of research on this intriguing molecule.

## 9. Conclusions

HCC is a fatal disease because the current treatment provides marginal benefits to patients in advanced stages [12]. There is a dire need to better understand the pathogenesis of this disease process, identify key molecules regulating HCC, and develop targeting strategies. As an oncogene, AEG-1 is a crucial regulator of all cancers studied so far. In HCC, it has more importance because it regulates the chronic inflammatory events that lead to HCC development [48,81,82]. As such, AEG-1 inhibition can be both a preventive and therapeutic approach for HCC. Developing appropriate combinatorial therapy involving AEG-1 inhibition might provide significant survival advantage to HCC patients. Immunotherapy is the first line of treatment for HCC [12], and AEG-1 inhibition synergizes with immunotherapy in breast cancer [76], thereby establishing the premise of testing this approach for HCC. AEG-1 and MYC cooperate to generate metastatic, aggressive HCC [113]. A selective and specific MYC inhibitor has been generated which exhibits a strong in vivo anti-cancer property, and synergizes with immunotherapy without exerting toxicity [170]. The combination of AEG-1 and MYC inhibition, along with immunotherapy, might be an effective way to counteract this virulent malady. AEG-1 expression levels might be used to stratify patients who might benefit from immunotherapy. Given AEG-1′s role in regulating the activation of macrophages [82], which play a central role in regulating HCC [171], inhibiting AEG-1 in both HCC cells and tumor-associated macrophages (TAMs) using targeted nanoparticle-delivered AEG-1 siRNA might be a potential approach for HCC treatment. HCC, diagnosed at early and intermediate stages, is amenable to treatment. However, the lack of an efficient blood biomarker prevents early diagnosis. The utility of an anti-AEG-1 antibody [147] as a potential early diagnostic marker needs to be analyzed in a clinical trial with a large cohort of patients. Future studies can explore this diverse array of mechanistic and translational research for a comprehensive understanding of this crucial oncogene.

## Figures and Tables

**Table 1:** Proteins and RNAs interacting with AEG-1 in HCC cells.

Molecule	Location of Interaction	Consequence	Reference
SND1	Cytoplasm	1. Stabilization of SND1 2. Augmented RNA-induced silencing complex (RISC) activity resulting in increased degradation of tumor suppressor mRNAs	[73,74]
RXR	Nucleus	1. Inhibition of RXR-dependent gene transcription 2. Contributes to metabolic dysfunction-induced steatohepatitis (MASH) 3. Contributes to non-thyroidal illness syndrome (NTIS)	[48,66,116]
DDX17	Nucleus	1. Stabilization of DDX17 2. Increased EGFR and MEK/ERK signaling	[123]
ZBTB17/Miz1	Cytoplasm	1. Sequestration of AEG-1 resulting in inhibition of NF-κB activation 2. Inhibition of macrophage activation and inflammation	[124]
mRNAs encoding endomembrane proteins	ER membrane	1. Increased protein translation 2. Chemoresistance 3. MASH	[48,63,121]

SND1: Staphylococcal nuclease and Tudor domain containing 1; RISC: RNA-induced silencing complex; RXR: Retinoid X receptor; DDX17: DEAD-box helicase 17; EGFR: Epidermal growth factor receptor; ZBTB17: Zinc finger and BTB domain containing 17; ERK: Extracellular signal-regulated kinase; ER: Endoplasmic reticulum.

**Table 2:** Potential clinical application of AEG-1 relevant to HCC.

Strategy	Application	Reference
IHC	Correlation between AEG-1 levels with overall survival and recurrence as a measure of prognosis	[142]
IHC	Combination of AEG-1 and GPC3 levels as diagnostic markers for HCC biopsy samples	[146]
ELISA	Serum AEG-1 antibody levels as a diagnostic marker for HCC	[147]
Targeted nanoparticle-delivered AEG-1 siRNA	Treatment of HCC in combination with ATRA; Prevention of MASH	[48,148]
Locked nucleic acid-modified (LNA) antisense oligonucleotide (ASO) for AEG-1	Treatment of HCC in combination with anti-PD-1 antibody	[149]
Small molecules (C26-A6 and C19) inhibiting AEG-1/SND1 interaction	Treatment of HCC	[76,77,78]

IHC: Immunohistochemistry; ELISA: Enzyme-linked immunosorbent assay; GPC3: Glypican-3; ATRA: All-trans retinoic acid; PD-1: Programmed cell death 1.

## Abbreviations

The following abbreviations are used in this manuscript:

AAT	α1-antitrypsin
AEG-1	Astrocyte Elevated Gene-1
AFP	Alpha-Fetoprotein
AFP-L3	Lens culinaris agglutinin reactive Alpha-Fetoprotein
AKT	Protein kinase B
ALB	Albumin
APC	Adenomatous Polyposis Coli
ASO	Antisense Oligonucleotide
ATG5	Autophagy Related 5
ATRA	All-Trans Retinoic Acid
BCL2	B Cell Lymphoma 2
CAR	Constitutive Androstane Receptor
CDKN2A	Cyclin Dependent Kinase Inhibitor 2A
CM	Conditioned Media
co-IP	Co-Immunoprecipitation
CREBBP	CREB Binding Protein
CTBP2	C-terminal Binding Protein 2
CTLA-4	Cytotoxic T-Lymphocyte-Associated Protein 4
CTNNB1	Catenin Beta-1 (β-catenin)
CPEB1/CPEB3	Cytoplasmic Polyadenylation Element Binding Protein 1/3
ctDNA	Circulating Tumor DNA
DEN	N-nitrosodiethylamine
DDX17	DEAD-box Helicase 17
DIO1	Type I 5′-Deiodinase
DNL	De Novo Lipogenesis
Dox	Doxorubicin
DCP	Des-γ-carboxy Prothrombin
DSS	Dextran Sodium Sulfate
DPYD	Dihydropyrimidine Dehydrogenase
EAAT2	Excitatory Amino Acid Transporter 2
EGFR	Epidermal Growth Factor Receptor
ELISA	Enzyme-Linked Immunosorbent Assay
ER	Endoplasmic Reticulum
ERG	ETS-Related Gene
ERK	Extracellular Signal-Regulated Kinase
FAO	Fatty Acid β-Oxidation
FASN	Fatty Acid Synthase
FDA	Food and Drug Administration
FXR	Farnesoid X Receptor
GPC3	Glypican-3
GSK3β	Glycogen Synthase Kinase 3 Beta
HBV	Hepatitis B Virus
HCC	Hepatocellular Carcinoma
HCV	Hepatitis C Virus
HCQ	Hydroxychloroquine
HFD	High Fat Diet
HITS-CLIP	High-Throughput Sequencing of RNA Isolated by Crosslinking Immunoprecipitation
HSC	Hepatic Stellate Cells
ICC	Intrahepatic Cholangiocarcinoma
IHC	Immunohistochemistry
IKK	IκB Kinase
IL1B	Interleukin 1 Beta
IL6	Interleukin 6
K63	Lysine 63
lncRNA	Long Non-Coding RNA
LNA	Locked Nucleic Acid
LPS	Lipopolysaccharide
LSF/TFCP2	Late SV40 Factor/Transcription Factor CP2
LXR	Liver X Receptor
MAFLD	Metabolic Dysfunction-Associated Fatty Liver Disease
MAPK	Mitogen-Activated Protein Kinase
miRNA	MicroRNA
Miat	Myocardial Infarction Associated Transcript
Miz1	Myc-Interacting Zinc Finger Protein 1(ZBTB17)
mRNA	Messenger RNA
MTDH	Metadherin
MDR1/ABCB1	Multidrug Resistance Protein 1/ATP-Binding Cassette Sub-Family B Member 1
MYC	Myelocytomatosis Oncogene
NF-κB	Nuclear Factor Kappa-Light-Chain-Enhancer of Activated B Cells
ncRNA	Non-Coding RNA
NLR	Neutrophil-to-Lymphocyte Ratio
NLS	Nuclear Localization Signal
NTIS	Non-Thyroidal Illness Syndrome
NR1H3/LXR	Nuclear Receptor Subfamily 1 Group H Member 3/Liver X Receptor
NR1H4/FXR	Nuclear Receptor Subfamily 1 Group H Member 4/Farnesoid X Receptor
NR1I2/PXR	Nuclear Receptor Subfamily 1 Group I Member 2/Pregnane X Receptor
NR1I3/CAR	Nuclear Receptor Subfamily 1 Group I Member 3/Constitutive Androstane Receptor
PAX	Paired Box Gene Family
PAR-CLIP	Photoactivatable Ribonucleoside-Enhanced Crosslinking and Immunoprecipitation
PD-1	Programmed Cell Death Protein 1
PD-L1	Programmed Death-Ligand 1
PEG	Polyethylene Glycol
PI3K	Phosphoinositide 3-Kinase
PIP3	Phosphatidylinositol (3,4,5)-Triphosphate
PIVKA-II	Protein Induced by Vitamin K Absence-II
PRMT5	Protein Arginine Methyltransferase 5
PTEN	Phosphatase and Tensin Homolog
PXR	Pregnane X Receptor
RAR	Retinoic Acid Receptor
RASSF1A	Ras Association Domain Family Member 1
RBP	RNA-Binding Protein
RIPK1	Receptor Interacting Serine/Threonine Kinase 1
RISC	RNA-Induced Silencing Complex
RNA-seq	RNA Sequencing
RXR	Retinoid X Receptor
ScFv	Single-Chain Variable Fragment
SEPTIN9	Septin 9
SND1	Staphylococcal Nuclease and Tudor Domain Containing 1
SN	Staphylococcal Nuclease
SRC-1	Steroid Receptor Coactivator 1
ST	Spatial Transcriptomics
STAT3	Signal Transducer and Activator of Transcription 3
TACE	Transarterial Chemoembolization
TCF/LE	T Cell Factor/Lymphoid Enhancer-Binding Factor
TGF-β	Transforming Growth Factor Beta
TKI	Tyrosine Kinase Inhibitor
TNFα	Tumor Necrosis Factor Alpha
TR	Thyroid Hormone Receptor
TRAF2	TNF Receptor-Associated Factor 2
TS	Thymidylate Synthase
VEGF	Vascular Endothelial Growth Factor
VEGFR2	Vascular Endothelial Growth Factor Receptor 2
VDR	Vitamin D Receptor
Y2H	Yeast Two-Hybrid
YB1	Y-box Binding Protein 1
ZBTB16	Zinc Finger and BTB Domain Containing 16
ZBTB17	Zinc Finger and BTB Domain Containing 17
ZDHHC6	Zinc Finger DHHC-Type Palmitoyltransferase 6
